# Good Practice in Health Reporting – guidelines and recommendations

**DOI:** 10.17886/RKI-GBE-2017-013

**Published:** 2017-03-20

**Authors:** Dagmar Starke, Günter Tempel, Jeffrey Butler, Anne Starker, Christel Zühlke, Brigitte Borrmann

**Affiliations:** 1 Academy of Public Health in Düsseldorf; 2 Local Public Health Office of Bremen; 3 District Office of Berlin-Mitte; 4 Robert Koch Institute; 5 Governmental Institute of Public Health of Lower Saxony; 6 NRW Centre for Health

**Keywords:** GOOD PRACTICE, HEALTH REPORTING, GUIDELINES, RECOMMENDATIONS, PUBLIC HEALTH, GERMANY

## Abstract

Health reporting provides descriptions of the health of a population, analyses problems and demonstrates areas in which action needs to be taken in health care, health promotion and disease prevention. As such, it provides a rational basis for participatory processes and a foundation for health policy decision-making.

Good Practice in Health Reporting was developed by a working group that includes representatives from all levels of health reporting with the aim of strengthening the field at the local, federal-state and national level. The document sets out guidelines and recommendations that are intended to provide professional guidance for the creation of health reports. It makes 11 recommendations that address the ethical principles behind health reporting, the necessary framework, the selection of topics (the report’s focus), the foundation of the work undertaken (data quality), data preparation, analysis, interpretation and protection, as well as communications and quality assurance. The pilot version of the document was presented at the conferences of the German Society for Epidemiology (DGEpi), the German Society for Social Medicine and Prevention (DGSMP) and the Federal Association of Physicians of German Public Health Departments (BVÖGD) where it was discussed and subsequently revised. After further review, the guidelines were adopted by all of these institutions. Finally, Good Practice in Health Reporting is to be strengthened and developed further as part of a comprehensive review.

Version 1.0 of Good Practice in Health Reporting was approved by the boards of the German Society for Social Medicine and Prevention and the German Society for Epidemiology, as well as by the extended board of the Federal Association of Physicians of German Public Health Departments.

*December 2016*

## 1. Foreword

At the beginning of the 1990s, the institution which at the time was known as the Advisory Council for Concerted Action in Public Health Care called for the establishment of a system of health reporting in Germany. Since then, health reporting has established itself as a separate form of reporting.

Good Practice in Health Reporting is aimed at providing professional guidance for the creation of health reports and ensuring high-quality health reporting.

These guidelines and recommendations were put together by an interdisciplinary working group consisting of representatives from the various levels of health reporting and relevant professional bodies in Germany.

The guidelines have become necessary because the differences in the legal basis, the availability of human and financial resources as well as data in Germany have led health reporting to develop in a highly diverse manner at the local, federal-state and federal level. This also explains why politicians often have such different views of health reporting.

Good Practice in Health Reporting is aimed at providing recommendations with regard to the methodology, contents and normative-ethical aspects of reporting, and helping ensure high-quality health reporting.

### 1.1 History

The first draft of Good Practice in Health Reporting was drawn up in 2011 after a review and evaluation of existing guidelines in epidemiology and secondary data analysis. This review was aimed at determining the applicability of existing guidelines to health reporting; it took into account various factors aimed at ensuring high-quality reporting. The first draft of Good Practice in Health Reporting was presented to the annual meeting of the German Society for Social Medicine and Prevention (DGSMP), the German Society for Epidemiology (DGEpi), and the conferences organised by the Federal Association of Physicians of German Public Health Departments (BVÖGD) and the Federal Association of Dentists in the Public Health Service (BZÖG). This led to a pilot version that was then discussed again by these professional institutions as well as at a workshop on health reporting at the federal-state and national level in 2015. This process resulted in the adoption of Good Practice in Health Reporting by the DGEpi, DGSMP and BVÖGD. The remarks and suggestions that developed out of discussions with these professional institutions have been taken into account in the present document.

We would like to take this opportunity to thank the many colleagues who participated in the debate by providing constructive proposals.

### 1.2 What happens next?

This version of Good Practice in Health Reporting was approved by the people and institutions involved in the discussions that resulted in this document being drawn up. For evaluation purposes we would like to gather together experiences and opinions of health reporters and specialists who use Good Practice in Health Reporting. Please send any comments or suggestions for improvements to this document by **31 March 2018** at the latest to Dr. Dagmar Starke, lecturer in epidemiology and health monitoring, Academy of Public Health in Düsseldorf: starke@akademie-oegw.de.

When providing comments, it would be very helpful if you could state which guideline you are referring to and provide reasons to explain your recommended changes.

After the closing date, the working group will review the suggestions and, if necessary, revise Good Practice in Health Reporting before publishing a new version soon afterwards.

## 2. Preamble

Health reporting provides an interpretive description of the population’s health, analyses problems and highlights areas where action needs to be taken.

Due to the misuse of medical statistics during National Socialism, health reporting as a discipline has developed relatively recently in Germany. The Ottawa Charter provides an essential basis for health reporting. In addition to calls for different political fields to be integrated in order to reduce social inequalities in health, the Ottawa Charter places a particular focus on information concerning the health of the population. Finally, in its 1993 report, the Advisory Council for the Concerted Action in Health Care (now known as the Advisory Council on the Assessment of Developments in the Health Care System) proposed that a system of health reporting be established in Germany to provide fundamental data that would enable targeted resource allocation.

Health reporting is now anchored in public health service laws in most German federal states; this situation has strengthened health reporting in Germany, and it has now been successfully established in a number of municipalities and federal states. During the late 1990s, health reporting in Germany gained a reliable superstructure with the establishment of Federal Health Reporting at the Robert Koch Institute and the Federal Statistical Office.

### 2.1 The aims and tasks of health reporting

Health reporting provides information to politicians and the public about health, illness, health risks and mortality events for a spatially and temporally defined population. As a steering instrument in health policy, health reporting offers an empirical basis with which rationally justifiable political decisions can be made. Furthermore, it accompanies health policy processes and enables public participation. As such, it is embedded in a particular political discourse. Reporting systems at the local, federal-state and national level are subject to the respective legal and political frameworks.

This means:

► Health reporting provides a description of the health of the population; it takes into account the unequal social and regional distribution of health risks and potentials for disease prevention, and demonstrates areas where action needs to be taken at the national, federal and local levels.
► Health reporting acts as an important basis with which to plan disease prevention and health promotion strategies, and can be used to evaluate public health measures.
► Health reporting continually gathers information about the health of the population, identifies possible changes in health at an early stage, and, therefore, can be used to make prompt health-policy decisions.
► Health reporting is not only aimed at experts and decision makers from the fields of politics and administration; it also addresses the general public.
► Health reporting supports the process of public opinion formation by providing information to the public and enabling them to participate in drawing up health policy objectives.
► Therefore, health reporting involves the civil society issue of public participation.

### 2.2 The methodological and theoretical foundations of health reporting

Health reporting needs a broad range of data. Ideally, it requires valid data that has been gathered in a uniform, standardised manner. Health reporting acquires data from its own specially conducted studies, the public health service, official statistics and process-generated data from other institutions in the health system (secondary data). The expertise of the people who collect the data should also be taken into account during the interpretation of the results, and, if appropriate, in the formulation of recommendations. Finally, it is essential that the efforts of data collection and the willingness of the people responsible to make their data available for health reporting is appropriately recognized.

In some areas, health reporting needs more data than is provided by the public health service and secondary data sources. Therefore, it is important that health reporting also takes into account epidemiological studies and representative health surveys. These studies provide more information about the population’s health, health-related behaviour and health care.

Health reporting is typically based on an interdisciplinary approach, with epidemiology providing its primary methodological and scientific basis. However, it also incorporates theoretical concepts and empirical findings from the social sciences, medicine, social medicine, medical sociology, health economics, health care research, health system research and health evaluation research as well as other disciplines.

The focus of health reporting is interlinked with a diverse range of other reporting systems, such as social, environmental and educational reporting, a point that is becoming increasingly important in the field. Since levels of health and disease strongly correlate with socio-structural factors, health reporting also includes data on socio-structural factors. Due to the large overlaps and interdependencies between health and social reporting, it is impossible to strictly distinguish between the two fields, and they can be better described as producing synergies. Nevertheless, due to the objectives and tasks assigned to health reporting, it is essential that health reporting continue to develop independently and that the work conducted in this field is undertaken with the appropriate level of expertise.

### 2.3 The foundations for work in health reporting, its framework and necessary resources

Health reporting is a complex task that requires detailed knowledge and adequate human, temporal and financial resources. Staff who conduct health reporting must be adequately qualified and undergo regular training. An appropriate level of resources enables high quality health reporting, ensures its practice-relevance and expresses appreciation for those who carry out health reporting.

### 2.4 Good Practice in Health Reporting

The aim of Good Practice in Health Reporting is to provide professional guidance on the process of creating health reports and to highlight the importance of health reporting as a basis for rational policy-making. One focus is on the interpretation of results in terms of their relevance for public health as a basis for health policy decisions.

In some situations it may be necessary, or even essential, to make exceptions to these guidelines. Then it is vital to clearly state that this has been done and to state the reason/s for doing so. This ensures that any deviations from standard procedures remain in accordance with good practices in health reporting.

Good Practice in Health Reporting complements Guidelines and Recommendations to Assure Good Epidemiological Practice [1] and Guidelines and Recommendations on Good Practice in Secondary Data Analysis [2] by setting out additional, yet central, aspects of health reporting. It also makes reference to appropriate sections in these two documents that contain essential information for planning, preparing and conducting empirical studies and processing, analysing and interpreting the data that this process produces. For information on the use of cartography in health reporting, please refer to Good Cartographic Practice in Health Care [3]. Health reports that contain advice or recommendations about health also need to take into account the guidelines set out in Good Practice in Health Information [4].

## 3. Guidelines and recommendations

### Guideline 1 (Ethics) Health reporting must be carried out in accordance with ethical principles and preserve human dignity and human rights.

*Ethical principles are those defined within general human and civil rights.*

#### Recommendation 1.1

Results that point to specific problems among individual population groups should be published with the differentiation and objectivity that is expected of scientific studies.

#### Recommendation 1.2

Health reporting should take into account the lives and needs of different social milieus; discrimination should not occur. This applies to all phases of health reporting.

#### Recommendation 1.3

The indicators used to analyse health-related issues should meet ethical standards. Classifications and indices should be reviewed to ensure that they are not based on normative assumptions or tacit value judgements.

#### Recommendation 1.4

Health reporting should maintain academic distance. Health reporting should not be permitted to become the voice of interest groups. By providing objective, verifiable information, health reporting creates transparency.

### Guideline 2 (Framework) Health reporting requires a defined political and organisational framework and a legal basis at all political levels.

*The legal basis extended to health reporting should set out the requirements needed to meet scientific quality standards and specify the conditions and framework that are required to ensure a good standard of health reporting.*

#### Recommendation 2.1

In addition to ensuring sufficient time and financial resources, it should be guaranteed that the staff responsible for health reporting has the appropriate methodological and technical qualifications.

#### Recommendation 2.2

If health reporting is conducted by external contractors, legally binding arrangements should be made for the creation of health reports, for accessing and using data, and for supplementary special analyses and expert opinions. This also applies to cooperation agreements with scientific institutions.

### Guideline 3 (Public Health) Health reporting provides the data needed to make health policy decisions.

*Health reporting identifies areas in need of action and uses this information to develop professionally based recommendations. The aim is to improve the health of the population, while taking into account equal outcomes and equal opportunities.*

### Guideline 4 (Subject of the report) Health reporting uses data to support its depictions of current aspects of the health status of the population or population groups. It provides information about and analyses of health determinants, frameworks and other aspects relating to health.

*Consequently, health reporting involves the study of explicit, operationalisable issues. This provides the basis for the report design, the choice of the population under study, the data that provides the report’s basis, and the selection of methods used to collect and analyse data. Moreover, working in this manner makes it possible to evaluate the report’s range of applicability and to assess the time and expenditure required to produce it.*

#### Recommendation 4.1

When selecting topics, health reporting should take into account an issue’s timeliness, as well as its public health and policy relevance. It should state the aim of a report, the reason it is being produced, and its addressees.

#### Recommendation 4.2

When dealing with the issues at the focus of a particular health report, and in order to avoid redundancies and outdated hypotheses, the latest scientific research should be consulted. In addition, health reporting should integrate findings from other reporting systems such as social and environmental fields. This makes it possible to adequately interpret and properly classify the results gained from health reporting.

#### Recommendation 4.3

Both the selection of the population under study and the selection of the indicators chosen to represent the data must be justifiable in terms of the report’s focus.

### Guideline 5 (Working basis) Health reporting should be based on the best available data and take the latest scientific research into account.

*As such, health reporting needs access to socio-demographic, socio-structural and regionally differentiated data. Data collection should be subject to quality assurance.*

#### Recommendation 5.1

The data used should be reviewed in order to determine their relevance, representativeness and informative value. If secondary data are used, the source of the data should be named.

#### Recommendation 5.2

The selection of the indicators deployed and the expert literature used to interpret the results should reflect the latest scientific research, and take into account all aspects of the issue/issues in question.

#### Recommendation 5.3

In order to detect changes over time, continuously measured indicators should be used. Regional comparisons of health-related issues should be undertaken by using standardised indicators.

### Guideline 6 (Data processing) A detailed plan should be drawn up for the acquisition and storage of all data used in health reporting, for data processing, plausibility testing, coding and data provision.

*In this context, the recommendations Guidelines and Recommendations to Assure Good Epidemiological Practice, Good Practice in Secondary Data Analysis and Good Cartographic Practice in Health Care apply.*

#### Recommendation 6.1

The choice of primary data and the rules for data collection should be documented. The continuity of the data collection rules, the population to be studied and the legal requirements need to be verified.

#### Recommendation 6.2

If data is used that has already been prepared, evaluated or published elsewhere, the initial reason why the data was collected, the principles that governed data collection and the procedures that were deployed for data evaluation should be stated.

### Guideline 7 (Data analysis) Data analysis in health reporting should be carried out promptly using scientific methods. The raw data that provide the basis for the results should be kept in a fully reproducible form in accordance with freedom of information laws.

*In this context, Guidelines and Recommendations to Assure Good Epidemiological Practice and Good Practice in Secondary Data Analysis apply. This is particularly the case with the documentary requirements for calculations involving complex figures and indices.*

#### Recommendation 7.1

When data is analysed for health reporting established epidemiological variables and procedures should be used.

### Guideline 8 (Interpretation) The interpretation of results is also a responsibility of health reporting.

*All interpretations should involve a critical discussion of the methods, data and results in the context of available evidence.*

#### Recommendation 8.1

One of the primary tasks in health reporting is to evaluate results. This process should not be influenced by personal, political or financial interests.

#### Recommendation 8.2

Results should be classified in accordance with the latest scientific research. This includes taking into account health determiners that are essential to the issue in question and illustrating their importance for the development of the population’s health. Alternative interpretations of the results, where relevant, should also be included.

#### Recommendation 8.3

Any limitations to the transferability of results to other populations or periods should be described. If applicable, conclusions that could not be made due to a lack of data should also be stated. When interpreting trends or changes over time, the fact that the meaning of variables or their definitions can change also needs to be considered.

### Guideline 9 (Privacy) When data is used for health reporting, the applicable data protection regulations should be observed.

*If questions arise, the Data Protection Office should be contacted.*

### Guideline 10 (Communications) Health reporting is not an end in itself. It must compete with other socially relevant issues for public attention.

*Health reporting should arouse people’s interest. In order to do so, appropriate media, forms of representation and stylistic devices should be used.*

#### Recommendation 10.1

Health reporting should use clear language that the general population can understand, while adequately addressing the respective target groups.

#### Recommendation 10.2

Health reporting should deploy various reporting formats and use different forms of media that are tailored to the interests and the manners in which the respective target groups access information. In addition to print media, the results produced by health reporting should be distributed via digital media.

#### Recommendation 10.3

The products of health reporting should be designed in a way that is attractive and appealing. When designing and publishing health reporting products, cost effectiveness should be taken into account.

#### Recommendation 10.4

Health reporting proactively presents results to target groups, as well as to professionals, relevant stakeholders and interested members of the public.

### Guideline 11 (Quality assurance) A quality control review of all relevant instruments and procedures is essential in health reporting.

*Health reporting’s most important asset is its probity, and, subsequently, the trustworthiness of the results it provides. Therefore, quality assurance is an indispensable component of any health report. The scope of the quality assurance undertaken must be in reasonable relation to the overall costs incurred during health reporting.*

#### Recommendation 11.1

Quality assurance should be conducted for all instruments and procedures that were employed, ranging from data collection to the data used, calculations and interpretations, and to the formulation of the recommendations derived from this process.

#### Recommendation 11.2

Quality assurance should take place during all stages of health reporting. Suitably qualified third parties that are not otherwise involved should participate in quality assurance.

## List of criteria

### Preliminary Note

The following list identifies aspects that normally need to be considered when creating health reports. The relevance of each of the aspects in a specific case, however, depends on the aim and the object of a particular health report and its associated complexity. Nevertheless, authors of health reports should review the relevance of these points with regard to their particular report; if the questions listed are not applicable to the issue at hand, they can be ticked as ‘No/Irrelevant’.

**1. Scientific Work**

**Table:**

Scientific standards	Yes	No/Irrelevant
The following scientific standards have been taken into account during the creation of the report: › the subject has been clearly delineated › the scope of the report is suitable considering the available material and the focus (there are no redundancies and unnecessary data have been omitted) › the report has a logical structure that is created successively (the second step results from the first) › the sources of data and other information are clearly stated › methods are described in detail and are suitable to the data being used › the results are presented in a structured manner › the results are objective (they are neutral and described with the necessary critical distance) › the results are verifiable (the data are available so that the results can be reproduced) › the data and results are scientifically accurate and complete. Observations and findings are reproduced truthfully › premises and conclusions are clearly identified › data and results from other publications are cited correctly and scientifically › sources have not been chosen selectively	□	□

**2. Reporting System**

**Table:**

**a) Transparency of the contracting authority and author(s)**	**Yes**	**No/Irrelevant**
The report clearly identifies the contracting authority.	□	□
The authors are stated (alongside their position and institution, if relevant).	□	□
Possible conflicts of interest are made clear.	□	□
**b) Planning the report**	**Yes**	**No/Irrelevant**
With regard to its focus, the report has been compiled in a manner that …		
› is cross-departmental	□	□
› is interdisciplinary (cooperation between several scientific disciplines)	□	□
› is multi-professional (cooperation between several professional groups)	□	□
› is integrative (cooperation between several departments/offices/government agencies)	□	□
› includes the population (participation), for example, during its design, and the determination of requirements, etc.	□	□
› involves external experts	□	□
A review of the availability of financial and human resources has been conducted.	□	□
A schedule has been developed involving all relevant actors.	□	□
**c) Structure of the report**	**Yes**	**No/Irrelevant**
The health report is based on the following structure:		
› a table of contents	□	□
› a list of diagrams/tables	□	□
› a list of abbreviations	□	□
› a preface/introduction	□	□
› a summary that includes the following: the report’s contracting authority, the report’s objectives, the report’s target audience, the report’s central findings and recommendations.	□	□
› a background section/a section explaining why the report is needed, and, if relevant, the public health relevance of the report’s focus	□	□
› a section describing the data	□	□
› a section describing the methods	□	□
› a section presenting the results	□	□
› a section discussing the results	□	□
› recommendations (see 7.b – c)	□	□
The health report contains credit notes that list:		
› the author(s)	□	□
› the publisher	□	□
› the year of publication	□	□
› the place of publication	□	□
› a contact person	□	□
› the number of copies published	□	□
A contact address has been provided.	□	□
**d) Funding**	**Yes**	**No/Irrelevant**
The source of the health report’s funding is made clear.		
› Funding is provided from the public budget.	□	□
› Funding is (partly) provided from third-party financing (If so, by whom?).	□	□

**3. Style, Layout, Printing And Distribution**

**Table:**

**a) The report uses an understandable and appropriate style**	**Yes**	**No/Irrelevant**
The general population is able to understand the report.	□	□
This includes ensuring that…		
› target groups are adequately addressed.	□	□
› jargon is avoided wherever possible.	□	□
› ‘run-on’ and convoluted sentences are avoided.	□	□
› active instead of passive formulations are chosen.	□	□
› filling words are left out.	□	□
abbreviations are explained.	□	□
Translating the report into plain language is necessary/would be useful.	□	□
**b) Overall layout**	**Yes**	**No/Irrelevant**
The health report has a clear overall layout.	□	□
The health report uses the corporate design of the city, state or governmental administration.	□	□
**c) Printing**	**Yes**	**No/Irrelevant**
The health report is available in printed form.	□	□
There is a distribution list.	□	□
The health report can be ordered (by phone, online, by post, fax).	□	□
**d) Distribution**	**Yes**	**No/Irrelevant**
The health report is freely available on the Internet.	□	□
The health report is available on the Internet only after registration.	□	□
The online version of the health report provides readers with the opportunity to submit questions via a contact form.	□	□
The publication of the health report was announced in various forms of media.	□	□
The results are actively presented to addressees.	□	□

**4. Subject of the Report**

**Table:**

**a) Objective**	**Yes**	**No/Irrelevant**
The objective of the report has been explained clearly and justified.	□	□
Objectives of the report could include: › analyses of data on morbidity and mortality with respect to the relevant population › evaluation of health-related measures › a reappraisal of a current situation that endangers (has endangered) the health of the population addressing and analysing a specific issue, such as a disease › identifying factors negatively affecting the health of the population › providing the basis for policy advice, e.g. on initiating measures in health promotion › devising proposals that can be tested empirically		
**b) Population/Demographic data**	**Yes**	**No/Irrelevant**
The population on which the report focuses is correctly represented.	□	□
Depending on the subject of the report, the following elements could be relevant: › population (average population/population analysed on a specific date) › gender distribution › age distribution › youth to old-age ratio › history of immigration/immigrant roots: country of birth, or, in the case of children and adolescents, their parents’ country of birth date of immigration to Germany nationality › migration (internal/external) › population projections › birth rate › fertility rate › mortality rate › years of life lost › preventable deaths Note: all the significant values and their definitions can be found in the health reporting indicator set provided by the Permanent Working Group of the Highest State Health Authorities (Arbeitsgemeinschaft der Obersten Landesgesundheitsbehörden, AOLG).		
**c) Gender**	**Yes**	**No/Irrelevant**
Data evaluation was conducted for the whole population, but also according to sex.	□	□
The results were produced in a manner that takes gender into account, i.e., a review has been undertaken of the influencing and outcome variables and their possible gender dependency.	□	□
**d) Social status**	**Yes**	**No/Irrelevant**
Individual social status, defined by (school) education, occupation, occupational status and income have been considered.	□	□
The data are evaluated separately depending on social status and the results are reviewed in relation to social position and, if necessary, social inequality.	□	□
The social structure of a particular territorial unit has been taken into account.	□	□
In order to describe the economic situation of the population of the area under analysis, the following complementary indicators may be considered: › the proportion of unemployed people/people without an income › the proportion of recipients of unemployment benefit II › the proportion of people in marginal employment › the median income › the proportion of single mothers › the share of children in need due to Social Code Book II (SGB II)		
**e) Age**	**Yes**	**No/Irrelevant**
Age groups have been categorised in a manner that is appropriate to the issue at hand.	□	□
For comparisons of different areas, an appropriate form of direct age standardisation is used: › old/new European standard population › standard population of the Federal Republic of Germany in the last available year › local age distribution of the federal state in question	□	□
If the available data only covers the standard population, indirect age standardisation is used.	□	□
The advantages and disadvantages of using the selected standard population have been made clear.	□	□
**f) Stages of life**	**Yes**	**No/Irrelevant**
Depending on the issue covered by the report, the individual stages of life (childhood, adolescence, adulthood, younger/older age) are taken into account.	□	□
**g) Migration**	**Yes**	**No/Irrelevant**
Depending on the issue covered by the report, data on experiences of migration are taken into account (e.g. country of birth, parents’ country of birth, length of stay, native language, nationality and residency status).	□	□
**h) Inclusion**	**Yes**	**No/Irrelevant**
The needs of people with disabilities are adequately addressed.	□	□
**i) Chronological developments and trends**	**Yes**	**No/Irrelevant**
In order to track changes in health, temporal comparisons have been conducted.	□	□
In order to track changes in health, trends have been projected.	□	□
**j) Regional comparisons**	**Yes**	**No/Irrelevant**
In order to determine regional differences, comparisons are made using suitable, relevant indicators.	□	□

**5. Basis of the data; data quality**

**Table:**

**a) Data selection**	**Yes**	**No/Irrelevant**
Data selection is related to the issue focused on in the health report.	□	□
› The data are routine data from: official statistics (such as those on hospital diagnoses, causes of death, the severely disabled, those who are incapable of work, rehabilitation and pension statistics or statistics provided by nursing care insurers) registries (such as epidemiological cancer registries, myocardial infarction registries) the census the resident’s registry office › The data are from scientific studies. › The data are from surveys conducted specifically for the report. › The data are derived from other data sources/from other data holders.		
A review has been conducted to ensure that the data sources provide an appropriate means of answering the issues at hand.	□	□
**b) Accuracy**	**Yes**	**No/Irrelevant**
The possibility of statistical errors is stated in the health report and was taken into account while interpreting the data. This includes: › sampling errors (such as during selection) › distortions created by data collection (for example, due to legal regulations) › missing values › measurement errors (for example, due to variations in standardised tests) › errors during data processing	□	□
**c) Timeliness of the data**	**Yes**	**No/Irrelevant**
› The report uses the latest available data.	□	□

**6. Data evaluation**

**Table:**

**a) Number of cases**	**Yes**	**No/Irrelevant**
The absolute number of cases is specified.	□	□
Relative numbers of cases are specified, for example, they are defined as the number of cases occurring among 100,000 people.	□	□
The population at risk is defined for each issue (the population among which the cases originated and that is at risk of having the disease is defined).	□	□
The numerator and denominator are clearly defined: the numerator states the number of cases or events and the denominator states the population at risk.	□	□
**b) Proportions (without reference to a specific population)**	**Yes**	**No/Irrelevant**
Proportions (shares) are given that provide information about the distribution of health-related events.	□	□
For example, in the case of infant mortality, the number of deaths that occurred during the first year of life in relation to all live births is specified.		
**c) Rates (with reference to a specific population)**	**Yes**	**No/Irrelevant**
Rates are provided that deliver information about the frequency of health-related events, such as contact to a doctor, new cases of illness, deaths or births in relation to the population at risk.	□	□
**d) Epidemiological measures**	**Yes**	**No/Irrelevant**
The following epidemiological measures of disease frequency have been calculated:		
› prevalence/prevalence rate	□	□
› incidence/incidence rate	□	□
mortality/mortality rate	□	□
› lethality/death rate	□	□
The following epidemiological measures of effect have been calculated:		
› standardised mortality rate (SMR)	□	□
› standardised incidence rate (SIR)	□	□
› relative risk (RR)	□	□
› hazard ratio (HR)	□	□
› odds ratio (OR)	□	□
The following epidemiological measures describing trends have been calculated.		
› absolute risk difference	□	□
› relative risk difference	□	□
› attributable risk	□	□
› population attributable risk	□	□
**e) Health economic considerations**	**Yes**	**No/Irrelevant**
Health economic issues have been taken into account in terms of expenditure, costs and financing.	□	□
The following calculations have been taken into account: › cost of illness, for example, direct costs, indirect costs › health expenditure calculations, for example, expenditure in public health care according to expenditure type, facility, and cost carrier › operating figures from the public health service, such as the number of staff		
**f) Electronic processing and evaluation**	**Yes**	**No/Irrelevant**
Data have been processed and evaluated electronically and the software used was specified.	□	□
**g) Evaluation strategies**	**Yes**	**No/Irrelevant**
All steps undertaken during data processing and data analysis have been made clear and documented transparently (via a log book, program syntax).	□	□
The raw data set has been subjected to a plausibility check.	□	□
The plausible raw data set is available in its original form (no newly formed or recoded variables have been added to it).	□	□
The main conclusions are based on results that have been reviewed by at least one other person.	□	□

**7. Interpretation, conclusion, recommendations**

**Table:**

**a) Mapping problems**	**Yes**	**No/Irrelevant**
The report substantiates specific problems.	□	□
Problems are mapped out using objective, deliberative interpretations of the results. Alternative explanations are discussed.	□	□
**b) Recommendations**	**Yes**	**No/Irrelevant**
As part of the health report, the evaluation of the results leads to the development of recommendations in need of an urgent response.	□	□
In the formulation of these recommendations, critical distance has been maintained in order to counteract attempts to instrumentalise the results by interest groups.	□	□
Recommendations are formulated with a view to developing possible strategies for hazard prevention/risk reduction.	□	□
Recommendations are made in regard to preventive measures.	□	□
Recommendations consider opportunities for health promotion.	□	□
When formulating recommendations, the congruence between results and recommendations was taken into account.	□	□
**c) Evaluation of the implementation of recommendations**	**Yes**	**No/Irrelevant**
The health reporting framework includes the evaluation of the implementation of recommendations.	□	□
